# Supplementary material for: Photon quantum entanglement in the MeV regime and its application in PET imaging
Source: Nat Commun. 2021 May 11;12:2646. doi: 10.1038/s41467-021-22907-5 (PMC8113573; doi:10.1038/s41467-021-22907-5)
Supplement: Supplementary file 1 — Supplementary Information [file 41467_2021_22907_MOESM1_ESM.pdf]

Supplementary Information - Photon quantum entanglement in the MeV regime and its application in PET imaging

D.P. Watts et. al.

Supplementary Note 1: Definition of kinematic factors

The kinematic factors are defined as:

$$K_a = \frac{[(1-\cos\theta_1)^3+2]\cdot[(1-\cos\theta_2)^3+2]}{(2-\cos\theta_1)^3\cdot(2-\cos\theta_2)^3} \quad \text{and} \quad K_b = \frac{\sin^2\theta_1\cdot\sin^2\theta_2}{(2-\cos\theta_1)^2\cdot(2-\cos\theta_2)^2}, \qquad (1)$$

where θ_1 and θ_2 are the polar scattering angles of the two gammas as defined in Fig. 1 of the main paper.

Supplementary Note 2: Verification of QE-Geant4 with analytical theory

In this note we show analysis which confirms that the simulation outputs in the new QE-Geant4 simulation, are in agreement with the analytical entangled theory forming the basis of the simulation (i.e. equation 2 in the main paper). We also show our analysis which confirms that the standard Geant4 is in agreement with the analytical theory predictions[1] for a (hypothetical) non-entangled state.

We firstly define the enhancement factor (R) which is used in the comparison. From equation 2 in the main manuscript, the probability of two entangled γ Compton scattering with a relative azimuthal angle $\Delta\phi = \phi_1 - \phi_2$ (where ϕ_1 and ϕ_2 are the azimuthal scatter angles of photon 1 and

2 respectively) is given by:

$$P(\theta_1, \theta_2) = A(\theta_1, \theta_2) \cdot \cos(2\Delta\phi) + B(\theta_1, \theta_2). \tag{2}$$

where $A(\theta_1, \theta_2)$ and $B(\theta_1, \theta_2)$ are simply the K_a and K_b factors multiplied by the $\frac{r_0^4}{16}$ constant in equation 2. The enhancement factor (R) is defined as the ratio of the perpendicular ($\Delta\phi = \pm 90°$) and parallel ($\Delta\phi = 0°$ and $\Delta\phi = \pm 180°$) scattering probabilities.

$$R(\theta) = \frac{P_\perp(\theta)}{P_{||}(\theta)}. \tag{3}$$

In table 1 (A) in the publication of Bohm and Aharonov[1], the parallel and perpendicular scattering probabilities for entangled γ undergoing symmetric Compton scattering (i.e. $\theta_1 = \theta_2 = \theta$) are given by:

$$P_{||}(\theta) = 2\gamma(\gamma - 2\sin^2\theta) \quad \text{and} \quad P_\perp(\theta) = (\gamma - 2\sin^2\theta)^2 + \gamma^2, \tag{4}$$

where $\gamma = (k_0/k) + (k/k_0)$, with k_0 and k the wave numbers of the incident and scattered photons, respectively. The resultant enhancement factor is plotted as the blue line in Supplementary Fig. 1. Note that Pryce and Ward[2] and Snyder *et al.*[3] determined the enhancement factor with different formalisms yielding strictly the same values.

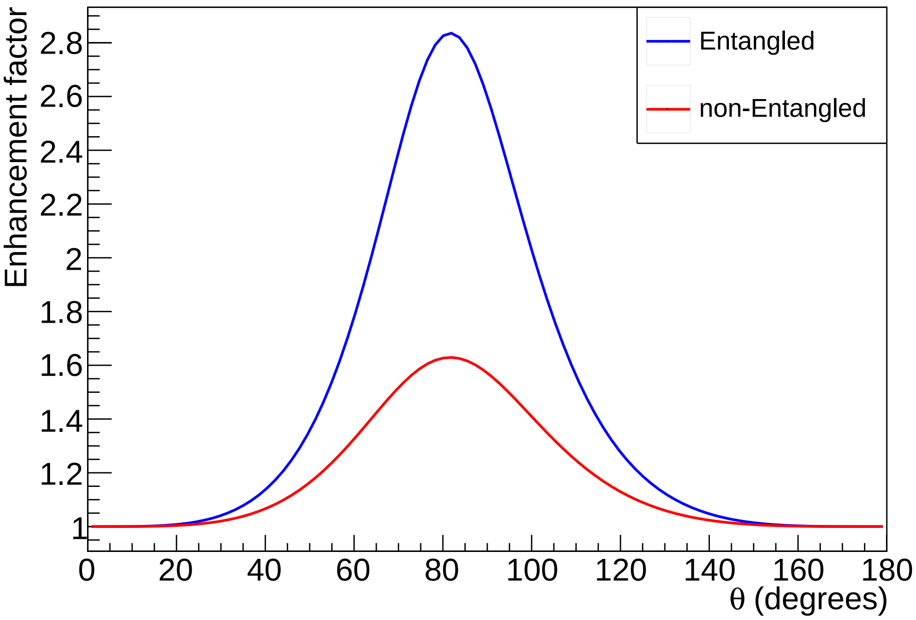
Entangled
non-Entangled
Enhancement factor
θ (degrees)
2.8
2.6
2.4
2.2
2
1.8
1.6
1.4
1.2
1
0
20
40
60
80
100
120
140
160
180

Supplementary Figure 1 Theoretical enhancement factors. Predicted enhancement factors (R) in double Compton scattering of positron annihilation gamma (see text) calculated using the theoretical formalisms of Bohm and Aharonov[1]. The enhancement is presented as a function of the common polar scatter angle θ for the scenario of symmetric Compton scattering of the two photons (i.e. $\theta = \theta_1 = \theta_2$). The blue curve shows the predicted θ dependence of the enhancement factor for the case of entangled γ. The red curve shows the prediction for a (hypothetical) state of orthogonally polarised, but non-entangled γ.

Similarly, line B_2 of Table 1 of Bohm and Aharonov calculates the parallel and perpendicular scattering probabilities for orthogonally polarised non-entangled γ as:

$$P_{\|}(\theta) = 2\gamma^2 - 4\gamma\text{sin}^2\theta + \text{sin}^4\theta \quad \text{and} \quad P_{\perp}(\theta) = 2\gamma^2 - 4\gamma\text{sin}^2\theta + 3\text{sin}^4\theta. \tag{5}$$

Note that a typographical error in Bohm and Aharonov for the latter term (reported $2\gamma^2-4\gamma^2\text{sin}^2\theta+3\text{sin}^4\theta$) has here been corrected. The predicted enhancement factors for this (hypothetical) non-entangled state are plotted as the red line in Supplementary Fig. 1.

The curves in Supplementary Fig. 1 illustrate the widely reported[2–4] maximum enhancement of 2.85 at θ=81.7° for this symmetric scattering case. Note that this is only achieved if the entangled treatment is employed. Neglecting the entanglement significantly reduces the maximum enhancement to 1.63, which is the proposed upper limit of the enhancement for a (hypothetical) non-entangled state[1].

In making comparison of the theory with the simulation the predictions must be integrated over a finite angular acceptance. Such acceptances are unavoidable in any Monte Carlo simulation predictions. To express the theory in a form in which such ranges can be included we combine supplementary equations 2 and 3 and solve for $\Delta\phi = 0°$ and $\Delta\phi = 90°$:

$$R(\theta) = \frac{B(\theta) - A(\theta)}{B(\theta) + A(\theta)} \tag{6}$$

which rearranges to:

$$\frac{A(\theta)}{B(\theta)} = \frac{1 - R(\theta)}{1 + R(\theta)}. \tag{7}$$

Thus, if we integrate supplementary equation 3 to obtain the enhancement factor over our polar angle range of interest ($R_{\Delta\theta}$) and take the normalisation $1/B$, we can calculate the relative double-scattering probability as:

$$\frac{P_{\Delta\theta}}{B_{\Delta\theta}} = \frac{1 - R_{\Delta\theta}}{1 + R_{\Delta\theta}} \cdot \cos(2\Delta\phi) + 1 \tag{8}$$

In Supplementary Fig. 2 we show the results of these calculations for the entangled and (hypothetical) non-entangled cases as the solid blue and red lines respectively. The theoretical predictions are integrated over the range ($67° \leq \theta \leq 97°$).

To obtain simulation predictions which could be compared directly with these analytic predictions we simulated the case of a "perfect" detector i.e. one having an effectively infinite detecting medium when compared to the path length of the photons. The perfect detector comprised an e^+ source placed at the centre of a $10 \times 10 \times 10$ m^3 cube of CZT. In characterising the Compton scatter processes for the two annihilation photons we used the exact properties of the incident and Compton scattered photons (accessible from the simulation).

Supplementary Fig. 2 shows the obtained $\Delta\phi$ distributions, from simulation, as the blue (red) data points for the entangled (non-entangled) scenarios respectively. Detailed agreement is observed between the QE-Geant4 simulation and the entangled theoretical prediction giving confidence in its correct implementation in the simulation. As expected, standard Geant4, which models all photon interactions as separable and non-entangled, follows closely the analytic predictions for double Compton scattering of a (hypothetical) non-entangled state by Bohm and Aharonov[1].

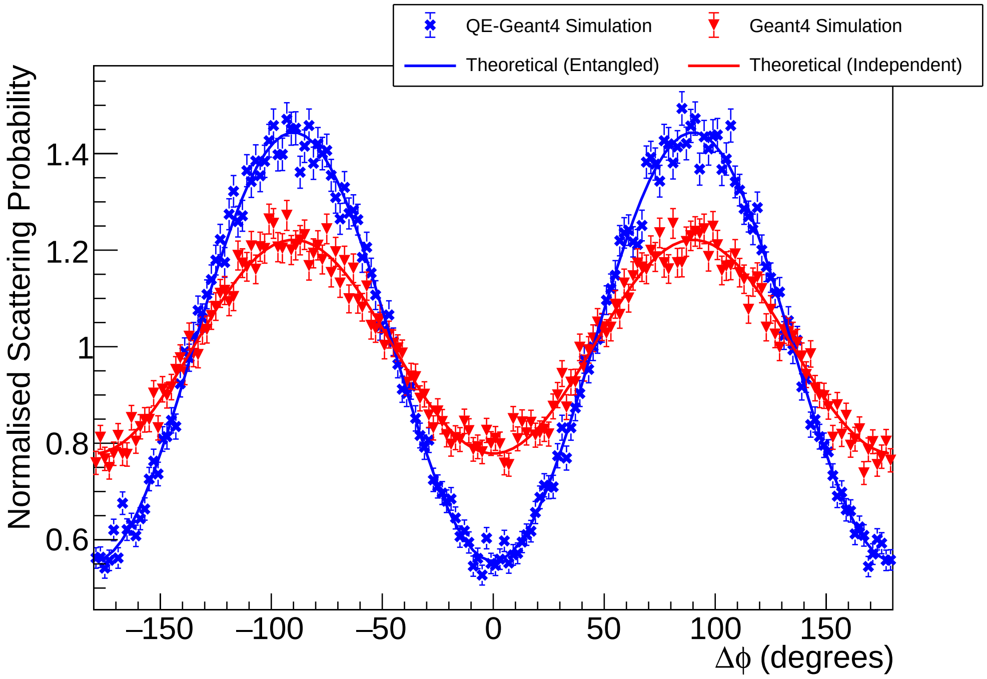

Supplementary Figure 2 Comparison of simulation with underlying quantum theory. The solid red (blue) lines show the analytical entangled[1–3] (non-entangled[1]) theoretical predictions of the probability for double Compton scattering of positron annihilation gamma. Both predictions have been integrated over the polar angle range for the Compton scattered photons of $67^\circ \leq \theta \leq 97^\circ$, using the formalism of supplementary equation

8. The predictions are presented as a function of the relative azimuthal scattering angle, $\Delta\phi$, between the two Compton scattered γ. The red (blue) data points, respectively, show the $\Delta\phi$ distributions predicted from QE-Geant4 (Standard-Geant4) simulations of an (effectively) infinite detector medium, with identical cuts on polar angular range. The error bars on the simulation data points represent the statistical errors (standard deviation) calculated from the number of events in each bin.

Supplementary Note 3: Further discussion of the validity of the entanglement formalism

The double Compton scattering (DCSc) entanglement witness was also exploited in early investigations of Bell's inequalities, providing evidence against (although not fully ruling out) hidden-variable theories in quantum mechanics[5–7]. Subsequently, optical EPR measurements proved more amenable for such tests due to the availability of more efficient linear polarisation analysers, which enabled tests with less auxiliary assumptions[8]. However, we remark that utilisation of the (experimentally verified) correlation in DCSc scatter planes in this work (equation 2), does not rely on the existence of loop-hole free EPR tests.

The small contribution of annihilation in flight, in which the positron annihilates before thermalisation, is neglected as assumed for previous Pa measurements[1,5–7,9–11] and only contributes in tissue at the 2% level e.g[12]. We neglect three-γ decays from ortho-positronium ($S = 1$) as they have a yield only 0.5% of the two-γ yield in PET[12], and could be further rejected by photon energy cuts. Initial ortho-positronium formation may lead to two-γ final states through singlet ($S = 0$)

pickup annihilation with an (external) electron in the surrounding medium e.g.[13]. We take such photons as having the correlated wavefunction of equation 1 (and DCSc cross section of equation 2) as implicit in previous measurements. This assumption is supported by the observation that, despite a range of positron thermalising media being employed, no deviation from equation 2 is observed in any previous experimental work on Pa[1,5–7,9–11] (or the current work).

We note that a very recent work[14] contradicted the original theory of Bohm and Aharonov[1] and the interpretation of all previous experimental works[1–3,5–7,9–11,15,16], by inferring a contribution to R from a hypothetical mixed state in Pa (in which the γ need not originate from the same annihilation event). The conclusion has already been refuted by Caradonna *et al.*[4] who re-derived $R = 1$ for mixed states in a matrix formalism in agreement with Bohm and Aharonov[1]. We note the unpolarised simulation presented in the current work (see main text and Fig 2) also supports that such events are consistent with $R = 1$. We therefore adopt the accepted theoretical interpretation of positron annihilation [1–3] in this work.

Supplementary Note 4: Additional statistical analysis of agreement between QE-Geant4 and experiment

In Supplementary Fig. 3, we present a bin-by-bin determination of the magnitude of the difference (the residual) between the experimental data and the simulation predictions presented in Fig. 2 of the main paper. The QE-Geant4 simulation (blue data points) matches the shape of the experimental data well over all $\Delta\phi$, with residuals distributed about zero. The standard Geant4 simulation

(red data points) however produces large residuals, failing to reproduce the amplitude of the experimentally observed $cos(2\Delta\phi)$ distribution. The χ^2/ν values in the comparison of experimental data to QE-Geant4 (experimental data to Geant4) are 1.87 (42.8).

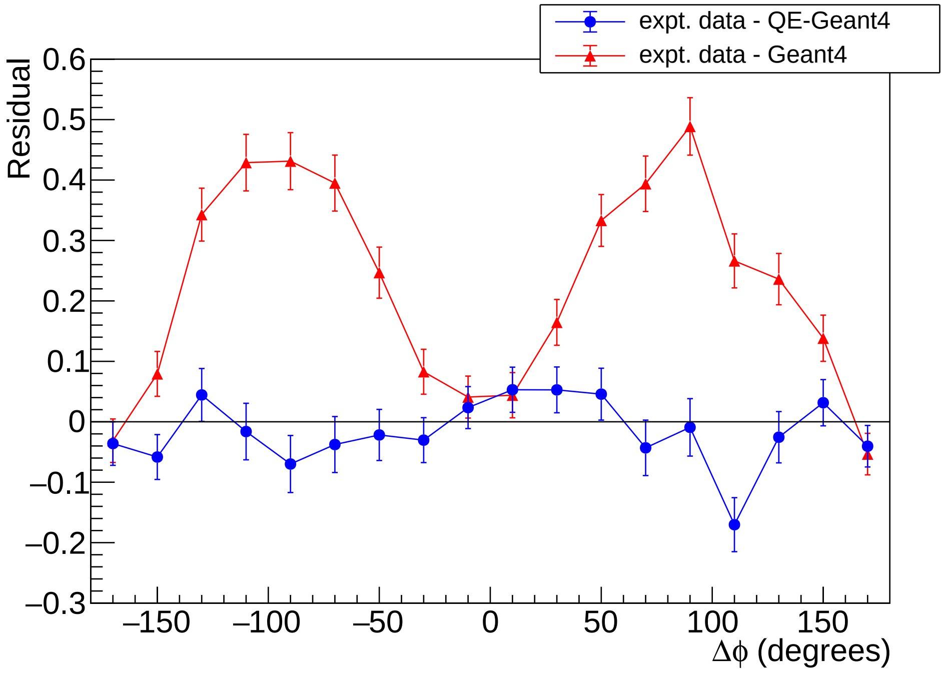

Supplementary Figure 3 Residuals in comparison of experiment with simulations. The blue data points show the bin-by-bin residuals between the experimental data from the Cadmium Zinc Telluride (CZT) PET demonstrator apparatus and the predictions of the QE-Geant4 simulation. The red data points show the residuals between the same experimental data but with the standard Geant4 simulation. Both the experimental data and simulation predictions in each bin are as presented in Fig. 2 of main paper viz. polar scatter angles of the gamma in the range $70^{\circ} \leq \theta \leq 110^{\circ}$ and a summed energy of the two clusters in each CZT detector in the range 480-530 keV. The (standard deviation) error

bars on the data points are the quadrature sum of the statistical errors for the experimental data and the simulation in each bin. The lines connect the data point centres to improve the clarity.

Supplementary Note 5: Details of the adopted QE-PET method

In this supplementary note we give more detailed description of the methodology adopted for QE-PET. As evident from the entangled theory for DCSc (equation 2 in main paper) and confirmed experimentally (Fig. 2), the true coincidence count rate varies as a function of the relative azimuthal scattering angle $\Delta\phi$. The discussion of the method below will be for the case of measuring true events with a background of scatter events, although as will be shown later the method also applies to the case of random backgrounds.

For a given bin of $\Delta\phi$, the total number of DCSc coincidences in the bin can be considered as the sum of the true and scattered events, i.e. $T(\Delta\phi) + S(\Delta\phi)$, as illustrated in Supplementary Fig. 4. In a given bin of $\Delta\phi$, the total number of coincidences can be expressed as a linear sum of the mean number of trues and scatters, weighted by appropriate scaling factors. For the two bins exploited in the current work ($|\Delta(\phi)| = 90°, 0°$) the total number of coincidences, denoted P_{90} and P_0 respectively are given by:

$$P_{90} = t_{90} \cdot \bar{T} + s_{90} \cdot \bar{S} \quad \text{and} \quad P_0 = t_0 \cdot \bar{T} + s_0 \cdot \bar{S}, \tag{9}$$

where $t_{90} = T(90°)/\bar{T}$ and $s_0 = S(0°)/\bar{S}$ etc. These latter terms account for the small residual $\Delta\phi$ dependence for the scatter coincidences. From our fits to simulation, this residual enhance-

ment for scatter coincidences arising from any remaining correlation in the polarisation planes of the detected γ is $R = 1.207$, compared to 2.541 for the true coincidences. Combining the two supplementary equations of 9, we can express the mean scatter contribution, $\bar{S}$, purely in terms of a linear combination of P_{90} and P_0, i.e.

$$\bar{S} = \frac{t_{90} \cdot P_0 - t_0 \cdot P_{90}}{t_{90} \cdot s_0 - s_{90} \cdot t_0}. \tag{10}$$

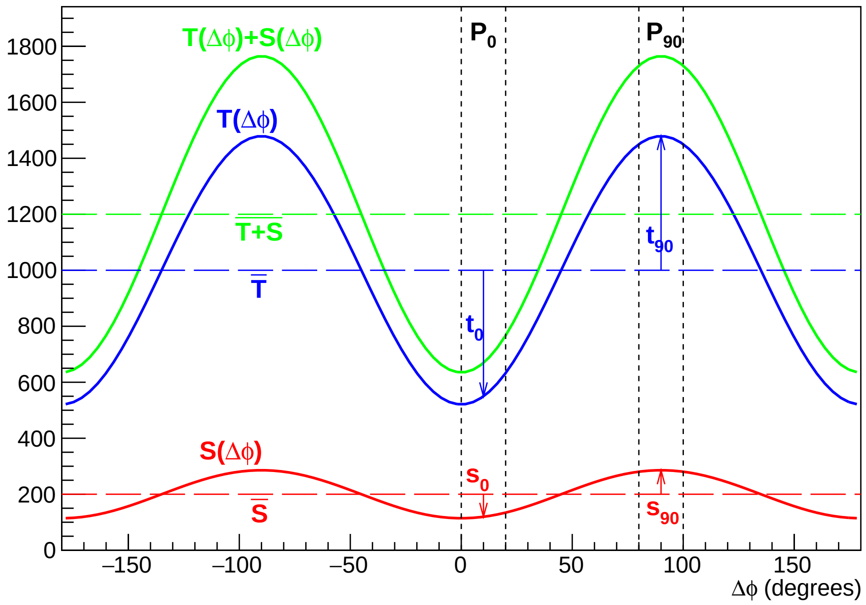

Supplementary Figure 4 Illustration of the method to extract the scatter contribution. A schematic showing the different contributions to the yield of double Compton scatter events in PET, presented as a function of $\Delta\phi$, the difference in the azimuthal Compton scatter angle of the two photons. The total of the measured coincidences is shown by the green curve. The blue curve shows a hypothesised fraction of true coincidences and the

red curve a hypothesised fraction of scattered coincidences. The y-scale is arbitrary, and the relative number of scatter events and the amplitude of their cos$(2\Delta\phi)$ dependence have been exaggerated for illustrative purposes. The variables used to extract the scatter contribution from a combination of two $\Delta\phi$ slices are indicated by the labels on the figure.

The coefficients s_0, s_{90}, t_0 and t_{90} are extracted from fits to simulated QE-Geant4 data. If for P_0 and P_{90} we use intensity profiles extracted from images generated with different cuts on $|\Delta\phi|$, we can use this methodology to extract a profile containing only the scatter contribution to the profile. A similar method can be applied to extract the true coincidences. The process for extraction of random background is identical, except that the coefficients s_0 and s_{90} are set to unity as the random coincidence count rate is independent of $\Delta\phi$.

Supplementary equation 10 highlights that this method is independent of the relative numbers of true and scatter coincidences; this information is contained within the two $\Delta\phi$ slices. The only input required is the dependence of these components on $\Delta\phi$. For random events, this is constant while for scattered events there is a small residual $\cos(2\Delta\phi)$ modulation. In the results, we adopted global coefficients integrated over the full phantom. To test this assumption, we compared the $\Delta\phi$ dependence in different regions of the phantom and found that the amplitude remained consistent within statistical uncertainties ($\sim 5\%$). Further checks were obtained from the extracted true and scatter contributions for profiles from different regions of the phantom, while using a single set of global coefficients for the residual scatter profile. The method was found to perform satisfactorily in all cases studied. As an example, in Supplementary Fig. 5 we present profiles through the middle

two capillaries. As was observed in Fig. 6, the agreement between the extracted and "actual" true profiles is excellent. The underlying shape of the scatter profile has different spatial dependence to that seen in Fig. 6c and is in good agreement with the "actual" scatter profile.

We should remark that the size of this small residual $\cos(2\Delta\phi)$ modulation is modelled assuming the expected collapse of the entangled state, as supported by the results presented in Fig 4. If future developments in our understanding of entanglement breaking at the MeV scale result in any changes in the form of this small residual $\Delta\phi$ dependence then the same QE-PET method with a modified treatment in the simulation would be applicable. We should also remark that in a real PET scan scenario, a combination of random and scatter backgrounds are present in the data. In applying the QE-PET methodology, their combined contribution would be quantified in the extraction. However, as the random coincidence rate can be determined independently (e.g. with the delayed window method[17]) then the contributions of scatter and random coincidences to the image could be separated if required.

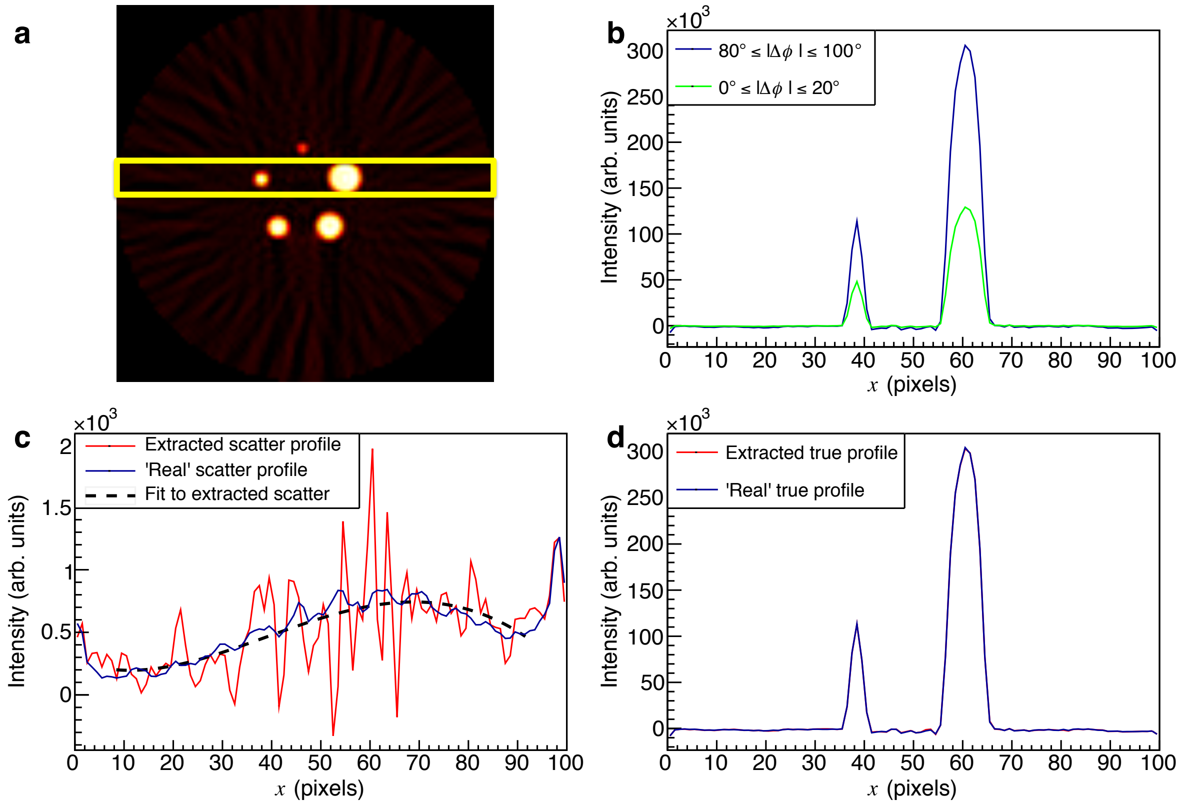

Supplementary Figure 5 Extraction of true and scatter contributions from middle capillaries. (**a**) FBP two-dimensional PET image of the NEMA-NU4 phantom for true events with a scatter background. (**b**) Intensity profiles through the region indicated by the yellow rectangle for different $\Delta\phi$ cuts, i.e. $0^{\circ} \leq |\Delta\phi| \leq 20^{\circ}$ (green), and $80^{\circ} \leq |\Delta\phi| \leq 100^{\circ}$ (blue). (**c**) QE-PET profile for scatter background events extracted from a scaled subtraction of the two $\Delta\phi$ cut profiles (red line). The blue line shows the profile from the scatter events in isolation using the information from QE-Geant4. The dashed line is a 4th order polynomial fit to the extracted scatter profile. (**d**) Profile extracted for true events with QE-PET (red line) compared to the profile of true events in isolation using QE-Geant4 (blue line).

Supplementary Note 6: Flow diagram illustrating the implementation of entanglement in QE-Geant4

Supplementary Figure 6 shows a flowchart of the QE-Geant4 event processing steps to include entanglement in the processing of the simulated events.

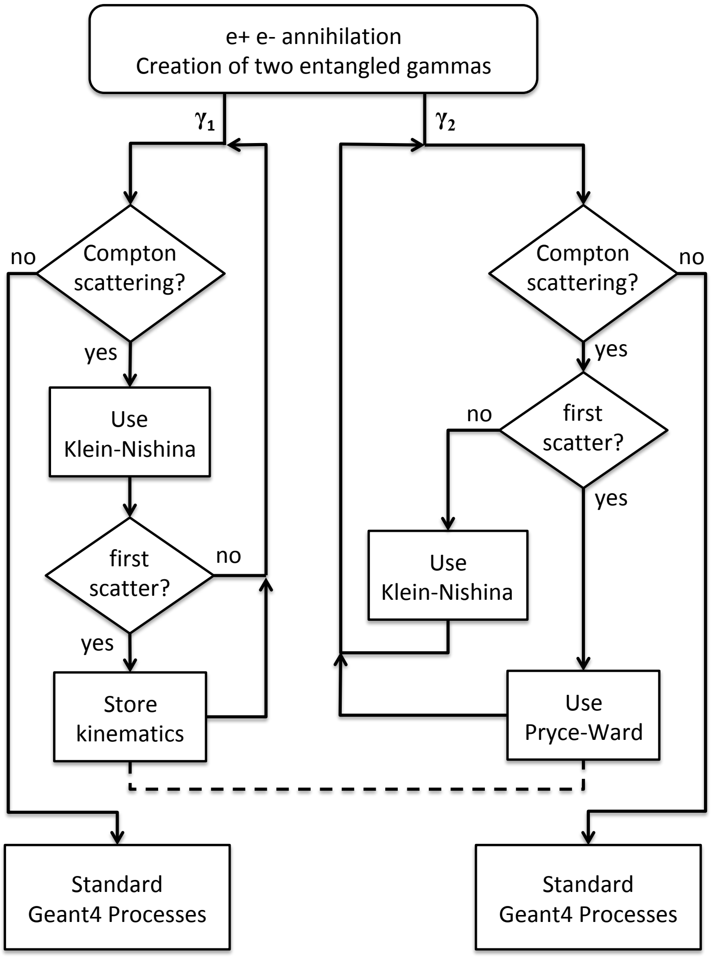

Supplementary Figure 6 Flowchart describing the implementation of entanglement in the simulation. The two gammas are processed sequentially, with γ_1 tracked

first. The kinematics of the first Compton scatter of γ_1 are stored and employed when calculating the first Compton scatter of γ_2. The "Use Pryce-Ward" action refers to employing the DCSc formula[2] (equation 2 of the main text) with the stored kinematics.

Supplementary references

1. Bohm, D. & Aharonov, Y. Discussion of experimental proof for the paradox of Einstein, Rosen, and Podolsky. *Phys. Rev.* **108**, 1070–1076 (1957).

2. Pryce, M. H. L. & Ward, J. C. Angular correlation effects with annihilation radiation. *Nature* **160**, 435 (1947).

3. Snyder, H. S., Pasternack, S. & Hornbostel, J. Angular correlation of scattered annihilation radiation. *Phys. Rev.* **73**, 440 (1948).

4. Caradonna, P., Reutens, D., Takahashi, T., Takeda, S. & Vegh, V. Probing entanglement in Compton interactions. *J. Phys. Commun.* **3** (2019).

5. Bertolini, G., Diana, E. & Scotti, A. Correlation of annihilation γ-ray polarization. *Il Nuovo Cimento B* **63**, 651–665 (1981).

6. Bruno, M., D'Agostino, M. & Maroni, C. Measurement of linear polarization of positron annihilation photons. *Il Nuovo Cimento B* **40**, 143–152 (1976).

7. Kasday, L. R., Ullman, J. D. & Wu, C. S. Angular correlation of Compton-scattered annihilation photons and hidden variables. *Il Nuovo Cimento B* **25 B**, 633–661 (1975).

8. Clauser, J. F. & Shimony, A. Bell's theorem. experimental tests and implications. *Reports on Progress in Physics* **41**, 1881–1927 (1978).

9. Wu, C. S. & Shaknov, I. The angular correlation of scattered annihilation radiation. *Phys. Rev.* **77**, 136 (1950).

10. Langhoff, H. Die linearpolarisation der vernichtungsstrahlung von positronen. *Zeitschrift fur Physik* **160**, 186–193 (1960).

11. Wilson, A., Lowe, J. & Butt, D. Measurement of the relative planes of polarization of annihilation quanta as a function of separation distance. *J. Phys. G: Nucl. Phys.* **2**, 613–624 (1976).

12. Harpen, M. D. Positronium: Review of symmetry, conserved quantities and decay for the radiological physicist. *Med Phys* **31**, 57–61 (2004).

13. Shibuya, K., Saito, H., Nishikido, F., Takahashi, M. & Yamaya, T. Oxygen sensing ability of positronium atom for tumor hypoxia imaging. *Commun Phys* **3**, 1–8 (2020).

14. Hiesmayr, B. C. & Moskal, P. Witnessing entanglement in Compton scattering processes via mutually unbiased bases. *Sci. Rep.* **9**, 8166 (2019).

15. Kasday, L. R. Experimental test of quantum predictions for widely separated photons. In d'Espagnat, B. (ed.) *Foundations of Quantum Mechanics: Proceedings of the International School of Physics "Enrico Fermi"*, 195–210 (Academic P XIV, 1971, 1971).

16. Faraci, G., Gutkowski, D., Notarrigo, S. & Pennisi, A. R. An experimental test of the EPR paradox. *Lettere al Nuovo Cimento (1971-1985)* **9**, 607–611 (1974).

17. Brasse, D. *et al.* Correction methods for random coincidences in fully 3D whole-body PET: Impact on data and image quality. *J. Nucl. Med.* **46**, 859–867 (2005).
